# How peer-activity leaders promote children’s well-being: perspectives from a CBPR-based multi-stakeholder study in Southern Sweden

**DOI:** 10.1080/17482631.2026.2758951

**Published:** 2026-11-04

**Authors:** Katarina Sjögren Forss, Gabriella Isma, Margareta Rämgård, Therese Sterner

**Affiliations:** a Department of Care Science, Faculty of Health and Society, Malmö University, Malmö, Sweden; b Citizen Health, Malmö University, Malmö, Sweden; c Department of Research, Development, Education and Innovation, Skåne University Hospital, Lund, Sweden

**Keywords:** CBPR, children, health promotion, leisure activities, peer-activity leaders, well-being

## Abstract

**Background:**

Adult role models and supportive environments are essential for children’s health, well-being, and development. Participation in organised leisure activities and access to local meeting places strengthen children’s social connectedness, safety, and resilience. In Malmö, Sweden, All-Activity Houses (AAHs) were established to promote children’s well-being through free and easily accessible activities led by peer-activity leaders who work closely within the children’s everyday environments.

**Objective:**

The aim of this study is to explore how peer-activity leaders perceive and promote children’s overall well-being, and to identify key mechanisms within the AAH context that contribute to these processes.

**Methods:**

The study applied a participatory action research design with a Community-Based Participatory Research (CBPR) approach. Data were co-created through multiple focus group meetings using a multistage focus group method within the CBPR teams, complemented by field notes from participant observations. Nineteen peer-activity leaders from four AAHs participated in the study.

**Results:**

Five themes emerged, reflecting how peer-activity leaders promote children’s well-being: building and sustaining trust; creating a safe place; applying flexibility in response to local conditions and children’s needs; promoting equality; and providing care.

**Conclusion:**

The findings suggest important mechanisms through which peer-activity leaders perceive themselves as contributing to children's well-being.

## Introduction

Adult role models and supportive social environments play a crucial role in shaping children’s health behaviours, development, and overall well-being (Atif et al., 2022). Research shows that children who have access to positive role models and mentoring relationships demonstrate better educational engagement, healthier behaviours, and improved psychosocial outcomes (Atif et al., 2022; Badura et al., 2015; Eime et al., 2013; Minkkinen, 2013). At the same time, children’s well-being is a multidimensional construct encompassing physical, mental, emotional, social, and environmental domains, which are deeply influenced by the contexts in which children grow up (Atif et al., 2022; Badura et al., 2015; Eime et al., 2013; Minkkinen, 2013).

Participation in structured leisure and after-school activities has consistently been associated with improved social-emotional skills, mental health, and resilience. Such activities provide safe environments, opportunities for meaningful relationships, and access to supportive adults and peers. These benefits appear to be particularly important for children living in socioeconomically disadvantaged areas, where access to safe spaces and supportive networks may be limited (Brock et al., 2018).

A growing body of research highlights the importance of peer-led approaches within such settings. Peer leaders may function as relatable role models, increasing engagement, trust, and the adoption of positive health behaviours through identification and social support (Badura et al., 2015; Christensen et al., 2024; Eime et al., 2013; Thoits, 2011; UNICEF, 1989). However, despite promising findings, there is still limited knowledge about how peer leadership is enacted in real-world community contexts and how it contributes to children’s well-being beyond specific outcomes such as physical activity (McHale et al., 2022).

In Sweden, the municipality of Malmö has addressed health inequities among children by establishing All-Activity Houses (AAHs) in socioeconomically disadvantaged neighbourhoods. These settings provide accessible, school-based (all school ages) after-school environments where children participate in activities co-created with and led by peer-activity leaders. These include both adult leaders and younger leaders in supporting roles; however, all activities are led by trained adult leaders who hold the main responsibility. The present study focuses on these adult peer-activity leaders. The AAH model combines structured activities with relational work, dialogue, and social support, aiming to promote children’s overall well-being and inclusion. Previous studies indicate that after-school environments such as AAHs may promote health, social cohesion, and resilience, particularly in communities with a high proportion of migrant families (Isma et al., 2023; Wagman et al., 2025). At the same time, research increasingly emphasises that children and community stakeholders are rarely involved in shaping such interventions, despite evidence that participatory approaches can strengthen relevance, trust, sustainability and resilience (Enskär et al., 2021; Isma et al., 2023; Ramji et al., 2020).

Community-Based Participatory Research (CBPR) offers an approach to address this gap by actively involving community members in the research process (Ramji et al., 2020; Wagman et al., 2025; Wallerstein, 2021). CBPR has been shown to enhance health equity, strengthen trust-based partnerships, and generate contextually relevant knowledge through co-creation with stakeholders (Rosa et al., 2025; Wallerstein, 2021). Within child and youth research, participatory approaches are increasingly recognised as essential for understanding lived experiences and designing effective, context-sensitive interventions (Rosa et al., 2025). Despite the growing interest in peer-led and participatory approaches, there remains a lack of in-depth knowledge about how peer-activity leaders themselves understand and operationalise their role in promoting children’s well-being within complex, real-life community settings such as AAHs. This represents an important knowledge gap, particularly in relation to holistic well-being, trust-building processes, and context-specific practices.

The present study is part of the Kids in Action for Health Equity (KAHEQ) programme, which applies a CBPR approach involving children, parents, peer-activity leaders, and researchers in socially disadvantaged areas in Malmö (Ramji et al., 2024).

The aim of this study is to explore how peer-activity leaders perceive and promote children’s overall well-being, and to identify key mechanisms within the AAH context that contribute to these processes. In the present study, overall well-being is understood as a holistic concept that includes children's physical, emotional, social, and psychological well-being, as well as their opportunities to experience safety, participation, supportive relationships, and a sense of belonging in everyday life. This broad understanding was used as a point of departure for exploring how peer-activity leaders perceive and promote children's well-being within the AAH context.

## Material and method

### Study design

This study applied a Participatory Action Research (PAR) design as the overarching methodological framework. PAR guided the study through its emphasis on participation, collective reflection, co-learning, and knowledge generation grounded in practice. Within this framework, a Community-Based Participatory Research (CBPR) approach was used to operationalise collaboration and equitable partnership between researchers and peer-activity leaders throughout the research process, ensuring that the study was grounded in local experiences, community priorities, and collaborative decision-making (Wallerstein et al., 2017). Thus, PAR provided the overall methodological orientation, while CBPR guided the practical implementation of stakeholder involvement and collaboration.

### Study context

The study was conducted within four AAHs located in socioeconomically disadvantaged areas in Malmö, Sweden: Lindängen (Lindängen School and Hermodsdal School), Rosengård (Apelgård School), and Heleneholm (Munkhätte School). These areas are characterised by limited socioeconomic resources and a high proportion of residents with migrant backgrounds. The AAHs provide structured after-school activities led by trained peer-activity leaders and function as local meeting places for children (Guldåker et al., 2021).

Several of the leaders had previously attended AAH themselves, although this was not the case for all. The leaders had diverse backgrounds and educational experiences; some had training related to children and child development (e.g., leisure-time pedagogy), while others did not. Recruitment primarily focuses on personal qualities and candidates’ motivation to work within AAH. Children participating in AAH were involved in the recruitment process and were given the opportunity to ask questions of potential candidates, based on questions they had developed together. All newly recruited leaders received internal training provided by AAH. Several of the leaders also came from the same areas as the participating children. Although the AAH model is a locally developed initiative in Malmö, comparable community-based and after-school programmes can be found both in other Swedish municipalities and internationally. However, there is limited published research examining these initiatives and their outcomes. These initiatives share a common ambition to provide safe environments, meaningful activities, and supportive adult relationships for children and adolescents, particularly in socioeconomically disadvantaged communities. At the same time, differences in organisational structures and local contexts should be considered when assessing the transferability of the findings from the present study.

### Participants and CBPR team

Peer-activity leaders employed at the AAHs for at least one year were invited to participate through purposive sampling. In total, 19 peer-activity leaders participated, together with four researchers from Malmö University, forming two CBPR teams (Figure 1). The researchers had prior experience of CBPR and qualitative methods, and their role was to facilitate the participatory process while ensuring methodological rigour. Written approval for the study was obtained from the Head of Unit at the Cultural Administration in Malmö City, and all participants at the four AAHs provided informed consent. Formal educational qualifications were not part of the recruitment criteria for this study. Instead, recruitment primarily focused on personal qualities, motivation, ability to build relationships with children, and commitment to the values and aims of the AAH model. Characteristics of the peer-activity leaders are presented in Table I.

**Table I.** Participants from the four AAHs.

AAH	Lindängen	Hermodsdal	Apelgård	Munkhätte
Peer-activity leaders (=*n*)	5	5	4	5
Gender	3 women 2 men	3 women 2 men	3 women 1 man	4 women 1 man
Age mean years (range)	42 (30–65)	30.8 (27–35)	26 (25–37)	33 (21–40)
Number of years at AAH mean (range)	4.6 (2–8)	5.4 (1–10)	3.0 (1–5)	4.8 (2–10)

**Figure 1.**
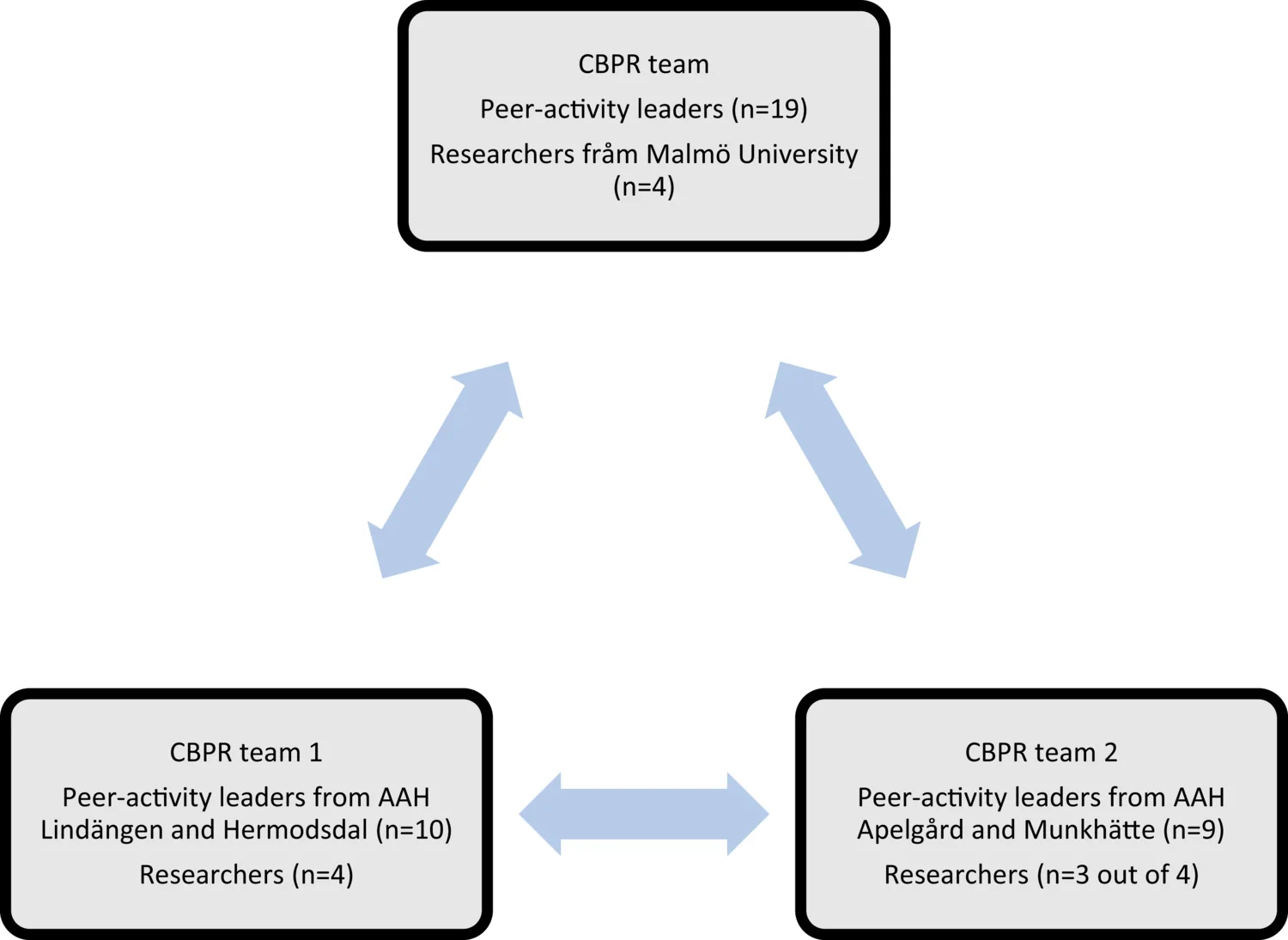
Structure of the CBPR team.

### Trust-building phase

This phase included repeated meetings and informal interactions, aiming to reduce power imbalances and create a safe environment for open dialogue. Previous research shows that partnerships based on trust can reduce power imbalances, improve the quality of the dialogue, and foster sustained engagement among co-researchers (Rich et al., 2024; Wagman et al., 2025).

### Data collection

Data were generated through: Multistage focus (MSF) group discussions, conducted over several repeated meetings and participant observations, documented through field notes. To ensure variation in the data, observations were selected in collaboration with AAH coordinators based on the planned activity schedules, enabling the researchers to observe a wide range of activities and everyday situations. The observations subsequently served as prompts for reflection and discussion during the multistage focus group dialogues. Data collection was conducted between spring 2023 and autumn 2024. All sessions were audio-recorded and transcribed verbatim.

#### Participatory process

The CBPR approach was operationalized through iterative cycles of planning, action, and reflection. Peer-activity leaders actively contributed to refining the study aim, shaping the research process, and interpreting findings. During the first two meetings, the CBPR teams discussed the study aim and how to proceed. At this stage, the participants preferred multistage focus (MSF) group dialogues and participant observations rather than traditional interviews. These preferences guided the design of the subsequent data collection.

The following meetings therefore consisted of MSF group dialogues based on observations of activities with children. The observations were initiated by the peer-activity leaders and followed by reflective group discussions. These meetings were structured to support open dialogue, shared decision-making, and mutual reflection, thereby fostering trust, strengthening partnerships, and helping to reduce power imbalances between researchers and peer-activity leaders.

The fifth and final meeting in each CBPR team focused on reflective dialogues related to the final analysis and interpretation of the data. In total, the two CBPR teams participated in ten meetings (five meetings per team), in addition to eight observation sessions. The meetings lasted approximately 60-120 minutes. Participation varied across meetings depending on the availability of the peer-activity leaders.

### Analysis

The data consisted of transcripts from the MSF group dialogues and field notes from participant observations. These data were analysed using thematic content analysis following Burnard et al. Both data sources were analysed using the same approach and integrated throughout the analytic process. The analysis was conducted in several iterative steps: (1) familiarisation with the data, (2) open coding, (3) grouping into categories, (4) development of themes, and (5) final refinement and validation.

First, all transcripts were read repeatedly by the researchers to gain an overall understanding of the data. Open coding was then performed, where meaning units relevant to the study aim were identified and labelled. The initial coding was conducted by the researchers, after which the emerging analysis was brought back to the CBPR teams for collaborative reflection and refinement. The codes were subsequently grouped into preliminary categories based on similarities and differences.

In the next step, the categories were compared, refined, and organised into broader themes representing key patterns in the data. The themes were continuously reviewed in relation to the original transcripts to ensure consistency and validity. When differing interpretations emerged, these were discussed within the CBPR teams and used to further explore the data rather than being treated as inconsistencies requiring resolution. Through ongoing dialogue across meetings, interpretations were progressively refined and developed.

In line with the CBPR approach, the analysis was conducted collaboratively within the CBPR teams. The peer-activity leaders contributed by reflecting on and discussing emerging codes, categories, and themes. Their input was used to validate and refine the interpretations, ensuring that the findings were grounded in their experiences. This iterative process of dialogue and reflection between researchers and peer-activity leaders strengthened the credibility of the analysis. Throughout the process, the researchers also reflected on their pre-understandings to minimise potential bias. Coding and theme development were conducted manually by the research team. Throughout the analytical process, ongoing reflexive discussions were conducted among the researchers and together with the CBPR teams to critically examine emerging interpretations and potential sources of bias. No formal audit trail or reflexive journals were maintained; instead, analytical decisions and interpretations were continuously discussed and refined throughout the study.

### Ethical considerations

This study was approved by the Swedish Ethical Review Authority as a part of an extensive research programme (Reg. No. 2023-00979-01). Participants received both oral and written information prior to participation. Verbal informed consent was obtained from all participants before participation in the study. At each MSF group discussion, participants were reminded of the study aims, their voluntary participation, and their right to withdraw at any time without consequences. Verbal consent was reaffirmed prior to data collection at each MSF group discussion and was documented through the audio-recorded research sessions.

## Result

Five themes emerged, considered to reflect how the peer-activity leaders promote children’s overall well-being. The key factors within the AAH context that contribute to the promotion of children’s well-being were thus identified. Although presented as separate themes, building trust, creating a safe place, and caring were highly interconnected in the peer-activity leaders’ narratives. Together, these themes describe complementary dimensions of a relational approach to promoting children's well-being. Trust appeared to function as a foundation for relationships, safety as a characteristic of the environment created within the AAHs, and caring as the practical expression of this relational work in everyday interactions.

### Building and sustaining trust

Trust was described as a foundational aspect of the peer-activity leaders’ work and something that permeated all activities, as well as all the AAHs, aimed at promoting well-being among the children. Relationships with children were built on trust, which required ongoing, intentional efforts. The peer-leaders emphasised that those efforts were a central part of their daily work and just as important as the activities themselves.

*We want the children to come and talk to us, and that’s only possible if there’s trust. It’s something we create together.* (Peer-activity leader 2)

The peer-activity leaders described how the children showed their trust in different ways, for example, by coming to them just to talk, often outside scheduled activities, about different issues ranging from school-related stress to family concerns. These spontaneous interactions were viewed as vital signs of a safe and trusting environment, and could be illustrated by the following excerpt from an observation:

*During the break, A (one of the peer-activity leaders) is out among the children. He ‘just sits’ there in the corridor and children constantly approach him. Many boys shake his hand and greet him. Some come several times (the same procedure, and A sees them each time). A few stop to chat, while others just walk by. A greets them as they pass by: ‘Hi XX’ (he knows everyone’s name). Everyone is seen and acknowledged. He moves between different places in the school. First the cafeteria, then the corridor, and then the playground. He is where the children are. He is consistently present and fully focused on the children (carries no phone).* (Observation 6)

The peer-leaders also stated that trust became even more important as children grew older. While younger children mostly came for the activities, older children, particularly girls, often attended only if a specific, trusted peer-activity leader was present. Some children were described as having special bonds of trust with a specific peer-activity leader, especially girls who experienced anxiety. Diversity among the peer-activity leaders was cited as a crucial element in fostering a supportive structure for developing trusting relationships with children. Furthermore, trust was mutual: the trust the peer-activity leaders placed in the children was evident, for example, during the recruitment of a new peer-activity leader. The children were involved in the process, being present during interviews and asking questions they had prepared themselves for potential candidates.

Trust could also be seen in the following excerpt from an observation:

*The session ends with five minutes of relaxation to calm music. L asks all the children to close their eyes. One child protests, saying that it might be dangerous not to see what’s happening around them. L assures them that she’s keeping an eye on everyone and that they can trust her. The child lies down and closes his eyes.* (Observation 3)

Moreover, the peer-activity leaders highlighted the importance of having a good and trusting relation with the caregivers, as their mission was to support the children’s well-being not only at school but also at home. Confirmation and seeing each child as an individual were described as central parts of the trust-building work. One aspect of this was calling the caregivers, at least a few times during the term, to tell them something positive their child had done, such as helping others or offering support. Caregivers were always very happy to receive these calls, and the children often told the peer-activity leaders that everyone at home was happy afterwards.

### Creating a safe place

The peer-activity leaders described the AAH as a place of safety, which was considered a crucial component of children’s well-being. They viewed themselves as embodiments of that safety, cultivating an environment where children could be themselves and create positive relationships.

*The things we do are so important and not always possible to put into words, but they [the children] trust us. We represent safety and the children also see how we are in the group and that we can laugh and have a good time together.* (Peer-activity leader 5)

The location of the AAH activities—within the school building and directly after school—was perceived as essential to promote safety, by both the children and their caregivers, as the school building itself was seen as a trusted and familiar place. Caregivers expressed their appreciation to the peer-activity leaders for the safe environment provided for their children, noting its positive effect on the children’s well-being.

Keeping the AAH open during school holidays was also described as crucial. A safe place might be even more important for children in vulnerable situations during the holidays, especially the long summer break, which in Sweden lasts approximately ten weeks. The peer-activity leaders emphasised that keeping the AAH open during holidays was important for maintaining stability for children who might otherwise lack daily routines or access to enriching activities.

### Flexibility in response to local conditions and children’s needs

A consistent theme among the peer-activity leaders’ narratives was the necessity for flexibility due to the different social and environmental conditions. While all four AAHs were situated in socioeconomically disadvantaged areas, the specific challenges they faced varied. Each area was described as unique, based on the conditions prevailing in that area, and consequently, the peer-activity leaders had to be flexible, as the same structure could not be applied at every AAH. One area was characterised by higher rates of crime and drug-related activity, prompting the peer-activity leaders to develop preventative and protective strategies tailored to those circumstances. Another area was more affected by child poverty, which led the AAH to buy mittens and beanies for eleven children at the school regardless of their participation in the AAH activities.

The peer-activity leaders contrasted their roles with those of the teachers. Whereas teachers had to operate within the constraints of a national curriculum, the peer-activity leaders stressed the importance of not having the same frames. That made them more flexible as well as available, with more opportunities to adapt to the children and to the current situation.

*But it’s almost impossible to stick exactly to a plan, given that a lot can happen and certain children may not like what we do. Then you have to adjust at very short notice.* (Peer-activity leader 8)

Flexibility also extended to how groups were structured in response to children’s expressed preferences. At one of the AAHs, the children wanted gender-segregated groups, especially as they grew older. In contrast, no such wishes had emerged in other AAHs, where even girls-only groups had not been requested.

The peer-activity leaders described this flexibility as a balance between, on the one hand, giving children opportunities to express their wishes, building trust, and listening to them, and, on the other hand, working towards greater equality.

### Promoting equality

The peer-activity leaders characterised the AAH as a platform for promoting gender equality, particularly in contrast to the inequalities that children may encounter in their home environments. For example, girls often had more responsibilities at home.

*The girls are expected to take on more responsibility at home compared to the boys, such as picking up siblings from preschool. Here, they are all allowed to be children.* (Peer-activity leader 1)

Efforts to promote equality were also described in terms of broadening the children’s horizons and providing them with opportunities to visit areas of the city or countryside that they had not previously experienced. The peer-activity leaders strived to keep these trips low-cost and accessible, often reachable by public transport, to ensure inclusiveness. They stated that there are many fun things to do in the surrounding area, but that someone must show it to the children as the caregivers are not very familiar with it. The peer-activity leaders emphasised that introducing children to new places expanded their understanding and awareness of their surroundings. It also helped to redefine these areas as safe and accessible, as the children trusted the peer-activity leaders and knew they would not take them to places that might be harmful. Children often returned to these places with their families, acting as ‘ambassadors’ who helped open up new experiences for their families. This not only boosted their self-esteem but also helped them grow in their caregivers’ eyes.

### Caring

Caring for the children was highlighted as central in the peer-activity leaders’ daily work to promote well-being. They described how some children conveyed that their caregivers were physically present but mentally absent. This was often because the caregivers were preoccupied with concerns about their country of origin and burdened with difficult and traumatic memories from both war and displacement, which could lead to shortcomings in their caregiving. The care at the AAH could be expressed in different ways; however, both in the discussions and during the field studies, the breakfast served every morning stood out as a typical moment of care that involved more than just the food.

*Food is a way of communicating, and we’re constantly talking over breakfast.* (Peer-activity leader 12)

Caring could also be seen in the following excerpt from an observation:

*The children come up to the leaders who are buttering sandwiches for them. While they’re buttering the sandwiches, the leaders have time to chat briefly with the children, asking if they’re tired, if they’ve slept well, etc. Each child is addressed by name. Some children come in but don’t want any breakfast; they just take something to drink. The leaders notice this and ask if they don’t want a sandwich, stressing the importance of having breakfast.* (Observation 8)

Due to limited economic resources, some children did not have breakfast at home, which was experienced as very shameful and something no one wanted to draw attention to. The peer-activity leaders described how they tried to handle this in a respectful and caring way. Caring was also described in terms of love, empathy, and confirmation, and the ability to meet the children where they were at the moment, whether they were feeling anger, frustration, sadness, or joy.

*There’s a lot of love in what we do and there’s empathy and closeness. We’re working from the heart.* (Peer-activity leader 17)

## Discussion

Trust was found to be a fundamental aspect of the peer-activity leaders’ work in promoting well-being among the children participating in activities in the AAH context. It can be described as an ongoing process that requires sustained efforts as well as commitment. Establishing trusting relationships with the children was regarded as a priority. It was also considered important to build trust with the children’s caregivers, reflecting an understanding of the importance of a holistic, joint, and collaborative approach to support the children’s well-being. Trust can be cultivated in diverse ways, something the peer-activity leaders not only appeared to understand but also actively applied in their daily work. The findings from the present study align with those from a recently published study within our research programme that aimed to explore key factors related to well-being from the children’s perspective (Burenby Yxne et al., 2025). Children in that study viewed peer-activity leaders as both reliable adults and supportive friends, underscoring their crucial role in building trust (Burenby Yxne et al., 2025). These findings are also consistent with international research highlighting the importance of relational approaches, trust, and adult role models in promoting children’s well-being in community and after-school settings (Badura et al., 2015; Christensen et al., 2024; Eime et al., 2013; Minkkinen, 2013; Thoits, 2011; UNICEF, 1989). Beyond confirming previous findings, the present study adds to the existing literature by illustrating how peer-activity leaders enact trust-building, flexibility, and relational work in everyday practice within a real-world community setting. This complements previous research, which has often focused on specific outcomes such as physical activity or programme effects, by providing a more nuanced understanding of the processes and relationships through which peer-activity leaders support children’s well-being in socioeconomically disadvantaged contexts.

Moreover, the City of Malmö demonstrates a trust-based governance approach by delegating responsibility to the peer-activity leaders, allowing them to design and implement activities within a loosely defined framework (Burenby Yxne et al., 2025; Statens offentliga utredningar, 2018). Such autonomy reflects distributed peer-activity leadership, where decision-making is shared and professional discretion is valued (Ball et al., 2023). When peer-activity leaders experience trust from their supervisors, it fosters a sense of psychological empowerment and professional confidence, which has been shown to enhance motivation and performance (Ball et al., 2023). This dynamic may help explain why the peer-activity leaders often go beyond formal expectations, showing increased commitment and caregiving in their work with children. An important aspect of our findings is the relative absence of expressed tensions or challenges in the peer-activity leaders’ accounts. While this may reflect genuinely positive experiences, it may also be influenced by contextual factors such as group dynamics, social desirability, or the collaborative nature of the CBPR process. Although participants were encouraged to discuss challenges and differing perspectives, such accounts were not prominent in the dataset. Therefore, the findings should not be interpreted as suggesting the absence of tensions or difficulties within the AAH context, but rather as reflecting the aspects of the work that participants collectively emphasised during the research process.

The peer-activity leaders’ approach to promoting children's well-being was characterised by a flexible application of the AAH concept, aimed at creating a sense of security for the children. Other studies on children’s perspectives on well-being have also identified flexibility as a significant factor (Burenby Yxne et al., 2025; Butler et al., 2022), as flexibility allows an adaptation to the specific conditions of each local context and to the evolving needs of the children involved. The flexible approach is responsive rather than prescriptive, enabling practitioners to tailor their work in alignment with both environmental factors and the lived realities of the participants (Carr-Fanning & Rihtman, 2025). At the same time, flexibility may involve navigating competing considerations. For example, peer-activity leaders described balancing children’s wishes for gender segregated activities with broader ambitions related to equality and inclusion. This illustrates that responding to children’s preferences is not always straightforward but may require ongoing negotiation between child-centred practices and wider social values. Notably, children are actively involved in all stages of the process, from the recruitment of peer-activity leaders to the selection and design of activities, as also shown in another recent study (Carr-Fanning & Rihtman, 2025). Such participatory involvement in contrast with conventional practices in Swedish schools, where decision-making is typically adult-led. It is an inclusive model that embodies principles of child agency and democratic participation and may be seen as an example of rights-based practice in line with the Convention on the Rights of the Child (Carr-Fanning & Rihtman, 2025; UNICEF, 1989). Similar flexibility cannot be provided by the formal school system, which is governed by national curricula and regulatory frameworks and rightly so, given its institutional mandate (Butler et al., 2022). In contrast, the AAHs operate under a different set of conditions, shaped by the children's specific needs and the characteristics of the local community. Their structure allows for a more adaptive and needs-based approach, where programming and peer-activity leadership are determined in close dialogue with the area's social realities. Previous research suggests that such approaches may promote well-being and resilience among children, including both those with and without supportive home environments (Mesman et al., 2021).

Although the peer-activity leaders rarely discussed structural inequalities explicitly, their descriptions of poverty, restricted opportunities, and unequal living conditions suggest that their everyday work involved responding to the consequences of broader social and economic inequalities.

Children look up to the peer-activity leaders and can identify with them, forming meaningful relationships rooted in trust and recognition. The peer-activity leaders are consistently present and adopt a caregiving role, meeting the children where they are emotionally, socially, and physically. Their availability throughout the school day, including breaks, on non-school days, ensures continuity of support, particularly when families are unable to provide such presence. This sustained relational engagement fosters a safe environment and positions the peer-activity leaders as reliable adult figures, often filling critical gaps in care and emotional availability (Ball et al., 2023). It also reflects principles of relational pedagogy and social scaffolding, emphasising the importance of adult attunement and proximity in promoting children’s well-being and development (Ball et al., 2023; Bellis et al., 2017; Butler et al., 2022).

The peer-activity leaders can be seen as brokers, facilitating connections and communications across different social contexts. They act as brokers between the children and the management of the AAH, ensuring that the children’s voices and needs are acknowledged in organisational processes. At the same time, they act as brokers between the children and the school, helping to bridge gaps in routines and support. Their role also extends to caregivers serving as a bridge between families and the school to foster trust, dialogue, and collaborative engagement. Through these brokering activities, the peer-activity leaders contribute to building trust and promoting inclusion, participation, and well-being within both educational and community contexts. This brokering role resembles what Rämgård and Avery (Rämgård & Avery, 2022) describe in their study of lay health promoters, who act as brokers in collaborative processes of networking between citizens and stakeholders in local meeting places in health promotion initiatives, a role that is well known in international contexts, but limited in Sweden (Rämgård & Avery, 2022).

In this regard, the present study adds to the literature by illustrating how similar brokering functions are enacted in a child- and community-focused setting. By mediating relationships across institutional and social boundaries, peer-activity leaders help create conditions that support children’s well-being, participation, and sense of belonging. This aligns with broader conceptualisations of well-being, such as those outlined in the WHO Geneva Charter, which emphasises the interrelationship between health, well-being, and supportive environments (WHO, 2021).

The findings should be interpreted considering the specific context in which the study was conducted. The AAHs operate in socioeconomically disadvantaged areas of Malmö and are characterised by a strong emphasis on participation, relational work, local adaptation, and trust-building. While the findings are not intended to be statistically generalisable, the mechanisms identified in this study may be relevant to similar community-based and after-school programmes that work to promote children's well-being through supportive relationships and meaningful participation. At the same time, transferability should be considered with caution, as differences in organisational structures, available resources, policy contexts, and sociocultural conditions may influence how such approaches are implemented and experienced in other settings.

Taken together, the findings suggest that peer-activity leaders contribute to children's well-being through trust-building, caring relationships, flexibility, equality-promoting practices, and meaningful participation. These mechanisms may be particularly important in community-based initiatives seeking to support children living in socioeconomically disadvantaged contexts.

## Strengths and limitations

The findings should also be interpreted in the light of several additional limitations. **First**, this study reflects the perspectives of peer-activity leaders and does not directly capture the experiences of children or caregivers. Although these perspectives have been explored in related studies within the broader research programme, their absence from the present study should be considered when interpreting the findings. **Second**, the collaborative nature of the CBPR approach, together with group dynamics and social desirability, may have influenced participants to emphasise positive experiences and successful aspects of their work. In addition, the organisational culture of the AAHs, characterised by trust, participation, and relational work, may have shaped how peer-activity leaders framed and discussed their experiences. **Finally**, the findings originate from one municipality and one specific intervention model, which should be considered when assessing transferability to other organisational and sociocultural contexts.

Due to the nature of the organisational structure, not all peer-activity leaders were able to attend every session. At times, other responsibilities or scheduling conflicts made them unavailable during our visits. Thus, the composition of the CBPR team was dynamic and changed over time, which presented challenges in maintaining consistency. As researchers, we needed to remain flexible and adapt to these circumstances. It was, furthermore, the peer-activity leaders themselves who requested that observations be conducted. This should be recognised as a strength, as it demonstrates their ability to influence the research process and reflects their active engagement in the CBPR teams. Moreover, it underscores their commitment to ensuring that we, as researchers, developed a genuine understanding of what key issues in the AAH context influence children’s overall well-being. We believe that this level of trust would not have been possible without the relationship-building processes that were fundamental to the CBPR team. The peer-activity leaders were also involved in the analysis process. Although we as researchers carried out the detailed analysis, the peer-activity leaders contributed by commenting and reflecting on the findings. Their input helped ensure that the analysis resonated with their experiences and that we used their language and perspectives.

## Data Availability

The data supporting the findings of this study are not publicly available due to the qualitative nature of the material and the risk of participant identification. The datasets consist of focus group discussions and field notes generated within a community-based participatory research project. Data may be available from the corresponding author upon reasonable request and subject to ethical approval and applicable data protection regulations.
